# Radiofrequency and microwave ablation as promising minimally invasive treatment options for papillary thyroid micro-carcinoma: a systematic review

**DOI:** 10.1308/rcsann.2025.0048

**Published:** 2025-07-24

**Authors:** I Chatzisouleiman, V Kolovou, N Tolley, G Mochloulis, M Katotomichelakis, K Chaidas

**Affiliations:** ^1^ Democritus University of Thrace - Medical School, Greece; ^2^ King’s College Hospital NHS Foundation Trust, UK; ^3^ Imperial College Healthcare NHS Trust, UK; ^4^ East and North Herts NHS Trust, UK

**Keywords:** Thermal ablation, Papillary thyroid microcarcinoma, Radiofrequency ablation, Microwave ablation, Systematic review

## Abstract

**Introduction:**

Ultrasound-guided thermal ablation has progressively emerged in the treatment of benign thyroid nodules. More recently, thermal ablation has also been proposed as a promising treatment alternative for papillary thyroid microcarcinoma (PTMC). This systematic review evaluates the efficacy and safety of ultrasound-guided radiofrequency ablation (RFA) and microwave ablation (MWA) for the treatment of PTMC.

**Methods:**

The PubMed and Cochrane databases were searched for relevant studies up to December 2023 following PRISMA guidelines. Volume reduction rate (VRR), complete disappearance rate (CDR), and associated complications following RFA and MWA were analysed.

**Findings:**

A total of 33 articles were included. Follow-up time ranged from 11 to 130 months. The efficacy of both MWA and RFA on PTMC was remarkable, with VRR reaching up to 99% at 12-month follow-up in the vast majority of patients, while CDR exceeded 80% in most studies. Following ablation, temporary complications at relatively low rates were reported including regional discomfort, voice change and haematoma, except for only one case with permanent recurrent laryngeal nerve injury.

**Conclusions:**

Ultrasound-guided thermal ablation of PTMC is a promising therapeutic approach for patients who are ineligible for, or refuse, surgery. Current research suggests that RFA and MWA provide favourable, low-cost outcomes, but larger multicentre, randomised studies are required to confirm the feasibility and safety of this new treatment modality.

## Introduction

Papillary thyroid carcinoma (PTC) is the endocrine malignancy detected most frequently worldwide. An ‘epidemic’ of papillary thyroid microcarcinoma (PTMC), referring to nodules ≤10mm, without capsular invasion, has been noted in recent years owing to the advance of medical imaging and strict screening policies. However, mortality rates of the affected population have not shown significant increases, raising questions about the aggressive overtreatment of low-risk PTMC.^1^

Thyroid surgery is considered as the standard therapeutic modality for PTMC, but often results in aggressive overtreatment, and is also associated with major complications that can increase morbidity.^2^ As most of these tumors remain unchanged in size, active surveillance (AS) has been an alternative management approach to low-risk PTMC.^3^ Despite the favourable results of AS, a high percentage of patients feel a psychological burden because of the diagnosis of cancer and seek an intervention.

Recently, a minimally invasive modality—ultasound-guided thermal ablation—has attracted attention as a new management strategy for PTMC. Thermal ablation includes three different techniques, radiofrequency ablation (RFA), microwave ablation (MWA) and laser ablation (LA), all of which are based on the method of applying high-energy heat that is transferred into the tumor via the insertion of a needle, without destroying the surrounding tissues.^4^ Thermal ablation is less invasive than surgery, cost-effective and can be performed on an outpatient basis. Several studies have demonstrated favorable outcomes for the treatment of benign thyroid nodules,^5^ but limited data regarding its application on PTMC exist. Thus, the routine use of thermal ablation as a therapeutic option for malignant thyroid nodules remains controversial. According to a recent meta-analysis,^6^ laser ablation seems to be less effective in the management of PTMC compared with RFA and MWA; hence, the latter techniques have recently gained popularity.

In this systematic review, we evaluate the efficacy and safety of RFA and MWA in the management of PTMC, including outcomes, complications, potential benefits and limitations of this novel treatment modality.

## Methods

A systematic literature review was conducted in accordance with the Preferred Reporting Items for Systematic Reviews and Meta-Analysis (PRISMA) guidelines.^7^

### Search strategy

A systematic search was performed on PubMed database and Cochrane library with the following keywords: ‘Papillary thyroid micro carcinoma’ OR ‘PTMC’ OR ‘Papillary thyroid carcinoma’ OR ‘PTC’ AND ‘Thermal ablation’ OR ‘Radiofrequency ablation’ OR ‘RFA’ OR ‘Microwave ablation’ OR ‘MWA’. Although no start search date was set, published literature up to 31 December 2023 was included.

### Eligibility criteria

The search strategy focused on the effectiveness of RFA and MWA in the treatment of PTMC, and associated complications. Eligible studies had to include patients with primary PTMC nodules (nodule size ≤10mm) without capsular invasion of the thyroid gland or lymph node metastasis (LNM). Only English language publications were included. Repetitive data, review articles, meta-analyses, case reports and letters to the editor were excluded. Studies with expanded PTC and insufficient data were also excluded.

### Quality assessment

To identify any flaws in the study design or conduct of the selected studies, and to ensure that the findings of the systematic review are transparent and accurate, we performed risk of bias analysis. Risk of bias assessments can help identify sources of bias, such as missing data, selection bias or issues around internal or external validity, competing interests or publication bias, and ultimately help inform clinical practice and policy-making. In this review, a Cochrane risk-of-bias tool was used to perform a risk-of-bias assessment for all the articles included.^8^ The domains assessed were related to the randomisation process, deviations from intended interventions, missing outcome data, measurement of outcomes and selection of the reported results. Finally, an overall judgement for each study was made following the Cochrane guidelines.

## Results

### Literature search results

**Figure 1:**
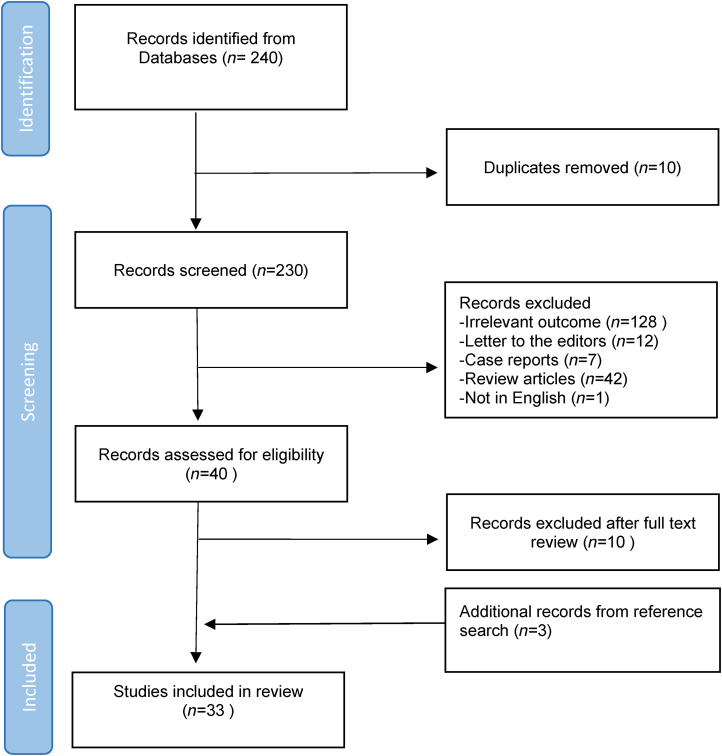
Flowchart of articles search and selection

The article selection and eligibility process is shown in Figure 1. A total of 240 articles were retrieved during the initial systematic literature search. Following the screening process, a total of 33 articles were finally included in the systematic review.

### Included study characteristics

Table 1 shows the characteristics of all included studies. The first study was published in 2014, with more studies following in the next years. Twenty-one retrospective, four cohort and eight prospective studies were identified, with the vast majority conducted in China (*n*=27). The majority of participants were female patients (75.4%). Patient age ranged between 14 and 81 years old. All studies included patients with solitary PTMC, except for one study that compared ablation on unifocal and multifocal PTMC, and one study with PTMC located bilaterally. A wide variation in follow-up time was noted, with a range between 11 and 130 months. Volume reduction rate (VRR), which was calculated as (initial volume–final volume)×100/initial volume at each follow-up assessment, complete disappearance rate (CDR) and associated complications of each study are presented in Table 2.

**Table 1:** Characteristics of included studies

Study	Year	Study design	Intervention	Country	No. of patients	Gender, m/f	Age range (mean±SD)	Follow-up time(months)
Yue *et al*^9^	2014	Prospective	MWA	China	21	6/15	29–81 (52.1±13.6)	11
Zhang *et al*^10^	2016	Prospective	RFA	China	92	23/69	14–69 (44.7±10.7)	18
Kim *et al*^11^	2017	Prospective	RFA	Korea	6	2/4	64–79 (72)	36–65
Yeong *et al*^12^	2018	Retrospective	RFA	Korea	6	2/5	36–83 (64±17)	15–24
Li *et al*^13^	2018	Retrospective	MWA	China	46	14/32	24–63 (43.6±9.3)	42
Teng *et al*^14^	2018	Prospective	MWA	China	15	6/9	27–59 (48.0±8.8)	36–48
Ding *et al*^15^	2019	Retrospective	RFA	China	37	8/29	26–77 (45.1±13.0)	16
Wu *et al*^16^	2019	Retrospective	RFA	China	198	57/141	22–65 (42.5±9.5)	24
Lim *et al*^17^	2019	Retrospective	RFA	Korea	133	19/114	19–79 (46±12)	39
Teng *et al*^18^	2019	Cohort	MWA	China	185	40/145	17–72 (42.2±11.7)	12–36
Yue *et al*^19^	2020	Prospective	MWA	China	119	27/92	24–81 (48.7)	12–101
He *et al*^20^	2020	Retrospective	RFA	China	95	24/71	55–74 (66±4.4)	36
Teng *et al*^21^	2020	Retrospective	MWA	China	41	13/28	27–63 (46.1±8.9)	60
Cho *et al*^22^	2020	Cohort	RFA	China	74	8/66	(46±12)	72
Zhang *et al*^23^	2020	Retrospective	RFA	China	94	24/70	(45.4±10.8)	64
Yan *et al*^24^	2021	Retrospective	RFA	China	47	10/37	23–63 (43.4±9.3)	12
Lu *et al*^25^	2021	Retrospective	MWA	China	73	13/60	16–74 (38.7±11.8)	12–24
Yan *et al*^26^	2021	Cohort	RFA	China	414	91/323	18–73 (43.6±9.8)	24–69
Wang *et al*^27^	2021	Retrospective	MWA	China	63	12/51	(43.6±14.2)	24
Cao *et al*^28^	2021	Retrospective	MWA RFA	China	673	147/526	22–81 (46 6±11)	6–60
Zu *et al*^29^	2021	Retrospective	MWA	China	320	83/237	18–76 (45.0±10.6)	6–88
Zhu *et al*^30^	2021	Retrospective	RFA	China	102	20/82	(43)	60
Cao *et al*^31^	2021	Retrospective	RFA MWA	China	725	152/573	22–81 (46±11)	6–60
Mauri *et al*^32^	2021	Prospective	RFA/LA	Italy	13	2/9	(49.3±8.7)	10
Song *et al*^33^	2021	Retrospective	RFA	China	112	18/94	22–66 (44.9±10.6)	13–60
Seo *et al*^34^	2021	Retrospective	RFA	Korea	5	0/5	29–51	130
Lim *et al*^35^	2022	Prospective	RFA	Taiwan	12	2/10	37–67 (51.2±11.0)	12
Zhou *et al*^36^	2022	Retrospective	MWA	China	44	Not reported	22–66 (44±11)	6–48
Yan *et al*^37^	2022	Retrospective	RFA	China	487	Not reported	Not reported	50
Zhang *et al*^38^	2022	Retrospective	RFA	China	157	42/113	(45)	18
Han *et al*^39^	2023	Prospective	MWA	China	1278	282/996	(44.3±10.7)	6–60
Yan *et al*^40^	2023	Retrospective	MWA RFA	China	474	104/370	(44.1±10.4)	77.2
Ren *et al*^41^	2023	Cohort	MWA	China	154	33/121	(38.4±10.7)	3–18

LA = laser ablation; m/f = male/female; MWA = microwave ablation; RFA = radiofrequency ablation

**Table 2:** Outcomes post thermal ablation, including nodule VRR, associated complications, PTMC recurrence, local neck lymph nodes metastases and complete disappearance rate

Study	VRR (%±SD)					Complications (*n*/*N* or %)	Recurrent PTMC	Cervical LNM	Complete disappearance rate (%)
	1-month follow-up	3-months follow-up	6-months follow-up	12-months follow-up	Last month follow-up				
Yue *etal*^9^	−0.28±1.42	0.48±2.86	0.91±0.13	–	0.90±0.14	Hoarseness (4/21) Choking (1/21) Cough (1/21)	0	0	22%
Zhang *et al*^10^	0.47±0.27	0.19±0.16	0.08±0.11	0.04±0.10	0	Hoarseness (4/92)	0	0	95.8%
Kim *et al*^11^	–	–	–	–	0.98±0.3	Hypertension with mild headache (1/6) Μild neck pain (1/6)	0	0	66.7%
Yeong *et al*^12^	–	–	–	–	0.47	Not reported	0	0	Not reported
Li *et al*^13^	−1517.37±1262.13	−550.19±539.67	−155.66±270.69	0.55±145.75	81.33±36.87	Neck pain-heating sensation (26/46) Neck swelling and discomfort (10/46) Voice change (1/46)	0	0	15.21%
Teng *et al*^14^	−2483.56±3804.43	−756.03±1070.46	−62.06±215.61	76.69±52.48	98.78±5.61	Burning sensation (5/15) Toothache (3/15 Hoarseness (1/15)	0	0	95.23%
Ding *et al*^15^	23.09±161.15	51.89±160.10	97.31±6.35	99.34±3.49	–	None (0/37)	0	0	100%
Wu *et al*^16^	73.9±13.7	90.5±8.2	96.1±5.9	98.8±3.2	99.8±1.0	Burning sensation (3/198) Hoarseness (5/198)	1	0	Not reported
Lim *et al*^17^	–	–	–	–	100	Hoarseness (1/133) Haematoma (2/133) First-degree skin burn (1/133)	15	0	91.4%
Teng *et al*^18^	−1770.85±2161.54	−534.58±758.92	−92.99±321.79	51.09±96.11	98.65±3.60	Hoarseness (5/185) Bleeding (11/185) Earache or toothache (21/185)	1	0	84.5%
Yue *et al*^19^	26.8±48.6	65.8±24.5	86.8±13.1	98.1±3.9	99.4±2.2	Haematoma (0.8%) Hoarseness (6.7%) Laboured breathing and coughing (3.4%)	1	1	93.9%
He *et al*^20^	−591.64±623.65	−170.89±319.51	9.74±128.43	77.99±45.26	99.78±1.54	Mild regional pain (1/95) Paroxysmal arrhythmia (1/95)	1	Not reported	100%
Teng *et al*^21^	−2913.88±3193.74	−10.75±1244.26	−297.19±583.75	−9.89±240.39	99.37±4.02	Hoarseness (2/41)	0	0	97.56%
Cho *et al*^22^	–	–	−202±787	54±138	100	Haematoma(2/74) First-degree skin burn (1/74) Voice change (1/74)	3	0	100%
Zhang *et al*^23^	–	–	–	–	–	Not reported	1	0	Not reported
Yan *et al*^24^	−511.78±569.06	−76.40±227.17	43.01±123.00	77.85±62.28	99.94±0.28	Local pain/discomfort (4/47)	2	1	92%
Lu *et al*^25^	−1226.35	−344.42	−29.66	80.28	100	None	3	3	77.78%
Yan *et al*^26^	−586.87±1072.61	−132.98±483.02	10.20±215.94	86.78±34.48	98.81±6.41	Local pain (16/414)	10	4	88.41%
Wang *et al*^27^	3442.23±4452.33	1408.29±1839.06	309.09±578.64	40.60±98.50	99.43±1.58	Burning sensation (19/63) Reactive hyperplastic lymphadenectasis (4/63) Local pain (63/63) Hyperthyroidism (9/63)	0	0	87%
Cao *et al*^28^	–	–	–	–	–	Hoarseness (14/673) Haematoma (3/673), Cough (1/673)	5	1	69%
Zu *et al*^29^	−1489.55±1936.55	−659.24±1345.55	−166.51±727.90	26.46±262.79	90.73±7.94	Neck pain (38/320) Hoarseness (12/320) Permanent recurrent laryngeal nerve injury (1/320)	0	6	60.3%
Zhu *et al*^30^	39	74	90	100	100	Hoarseness (2.9%)	0	2	100%
Cao *et al*^31^	–	–	–	–	–	Hoarseness (1.9%) Haematoma (0.6%) Cough (0.1%)	5	1	71%
Mauri *et al*^32^	–	–	–	–	–	Dysphonia and discomfort (3/11)	0	0	Not reported
Song *et al*^33^	–	–	–	91	100	Not reported	0	0	100%
Seo *et al*^34^	–	–	–	–	–	Not reported	0	0	60%
Lim *et al*^35^	−233	17.14	53.57	100	100	None	0	0	61.54%
Zhou *et al*^36^	–	–	–	–	–	Unspecified complications (4/44, 6.8%)	Not reported	Not reported	54%
Yan *et al*^37^	−538±973.71	−120.17±452.48	22.08±199.91	86.46±47.30	99.40±4.43	Not reported	11	5	95.61%
Zhang *et al*^38^	−88	43.02	83.65	99	99.98	Hypoparathyroidism (1/157) Voice change (3/157)	0	0	29.3%
Han *et al*^39^	–	–	–	–	–	Hoarseness (14/1278) Cough /choking (1/1278) Infection (1/1278) Haematoma (1/1278)	6	10	98.78%
Yan *et al*^40^	−431.3	−87.5	50	100	100	Dysphonia (8/474)	0	5	99.36%
Ren *et al*^41^	−1504.275	−461.983	−169.284	32.462	100	Hoarseness (4/154) Hypothyroidism (19/154)	2	5	100%

LNM = Lymph node metastasis; *n* = number of patients with complications; N = total number of patients; PTMC = papillary thyroid micro-carcinoma; VRR = volume reduction rate.

### Risk of bias assessment

**Figure 2:**
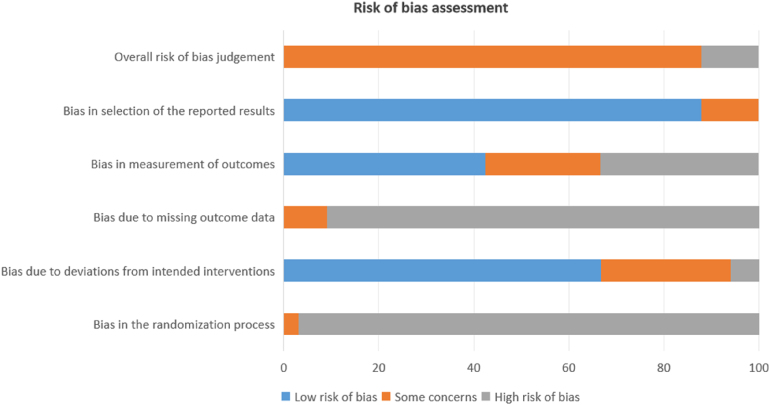
Cochrane risk of bias tool showing the proportion (%) of studies with low risk, some concerns or high risk of bias.

The risk of bias assessment findings indicated that all the studies included in this review had some concerns regarding risk of bias (Figure 2)*.* All 33 studies exhibited some concern or high risk of bias in the randomisation process and in the missing outcome data domains. In contrast, the other three domains generally demonstrated lower risk of bias levels: some concerns or a high risk of bias were found in 19 studies for the measurement of outcomes domain, in 11 studies for the deviations from intended interventions domain and in 4 studies for the selection of the reported results domain. High risk of bias in the randomisation process and in the missing outcome data was due primarily to the population samples used to perform the analyses, i.e, single-centre retrospective studies with very small study populations were often used. Furthermore, short follow-up periods were often insufficient to thoroughly assess the impact of the considered intervention. These features limited the generalisability of the study findings and their external validity.

### Thermal ablation principles

Since 2016, the use of radiofrequency waves has been proposed for the management of PTMC. RFA is based on hyperthermal energy that is transferred via electrodes to the targeted microcarcinoma, leading to irreversible damage to the cell membrane and coagulation of the cancerous tissue without causing injury of the surrounding anatomical structures.^4^ On the other hand, microwave technology uses electromagnetic waves to generate heat on a specific region during a short period of time, resulting in coagulation and destruction of the targeted nodule. After initial use for the treatment of benign thyroid nodules, MWA has recently been applied to PTMC cases as well.^4^

The diagnosis of PTMC was made following ultrasound-guided fine needle aspiration (FNA) and histological examination in all cases. Thermal ablation was performed under local anaesthesia in all studies, whereas some centres also performed hydrodissection with normal saline before ablation to minimise the risk of damage to the surrounding tissues. There was no significant variation in terms of technique details and mean duration power between the institutions publishing their data.

### Volume reduction and complete disappearance rate

Table 2 presents the rates of volume reduction of PTMC nodules following thermal ablation at the first, third, sixth and twelfth month of follow-up, as well as at the last month of each study’s follow-up. One month post thermal ablation, a temporary augmentation in nodal size was reported in 15 studies, followed by tumor shrinkage over the next months. The vast majority of included studies reported a VRR of 80-100% at the last month of follow-up. There was no statistically significant difference in volume reduction between RFA and MWA groups.^31^

Overall, a wide variation in CDR was noted, ranging from 15.21% to 100%. However, the most recent studies with larger samples and longer follow-up time reported significantly higher rates compared with the earlier studies. Most articles indicated favorable outcomes following RFA with a CDR over 95%, whereas two studies with a small number of patients noted a CDR of 66.7% and 61.5%, respectively.^11,35^ Similarly, most MWA studies reported a CDR higher than 80%, with a low rate noted in only one study.^13^ The latter was attributed to possible higher central temperature and superior carbonisation ability of MWA compared with the RFA method, making it more difficult for the ablated area to dissolve over time.

### Complications and quality of life

Both RFA and MWA showed similar complications, with mainly transient symptoms related to the thermal energy that is transferred via the ablation electrode to the anatomical region of the nodule. The most common complaints, as shown in Table 2, included burning sensation, mild regional neck pain, temporary voice change and local haematoma with spontaneous resolution within a few days. Other less frequent complications were fever, toothache, cough, paroxysmal arrythmia, transient hypoparathyroidism and transient hypertension. Among all studies, only one case with permanent recurrent laryngeal nerve injury after MWA was reported.^29^ No postoperative life-long hormonal replacement therapy was needed in any patient of the study groups. No life-threatening complications were reported during or after the procedure.

Patients undergoing RFA had better postoperative quality of life and complication rates, as well as better scar cosmesis compared with thyroidectomy.^23,38^ Recent studies with a higher number of participants and longer follow-up demonstrated promising outcomes with mild complications of hoarseness and regional pain with a rate that does not exceed 5% of patients.^28,38^ A comparative study with 5-year follow-up indicated significantly higher cost of hospitalisation, presence of major complications such as permanent recurrent laryngeal nerve injury and hypoparathyroidism, and worse quality of life in the thyroidectomy group compared with the RFA group.^23^

Likewise, comparative studies on MWA versus surgery also indicated better quality of life and lower postoperative complication rates in the MWA group.^13,27,29^ Namely, a comparative study by Li *et al* reported a complication rate of 4.3% in the MWA group compared with 32.6% in the surgery group, including permanent laryngeal nerve palsy (2.2%), permanent hypothyroidism (19.6%) and dysphagia (8.7%).^18^

### Recurrence

Most studies reported no recurrent PTMC or cervical metastatic lymph nodes detected after RFA (follow-up, 16–130 months) and MWA (follow-up, 11–42 months). There were no patients with distant metastasis reported. Overall, recurrent PTMC was detected in 1% of the patients, whereas 0.7% of the patients developed LNM. The vast majority of these patients underwent additional ablation with complete disappearance afterwards.^14,16,18,20,24–26,39,41^ Even in the case of patients with PTMC recurrence or LNM, a disease-free survival rate of 98.78% was reported during a follow-up of 60 months.^41^

## Discussion

The aim of this review was to explore a potential role for thermal ablation in patients with low-risk PTMC. The results reported, concerning the efficacy and safety of this treatment modality, were encouraging in a number of ways. First, significant VRR was noted in almost all studies; second, the complete disappearance rate exceeded 80%; third, the complications detected were mainly temporary and of minor severity, and were resolved in a short period of time; and fourth, there was no need for life-long hormonal replacement therapy after the procedure.

Currently, conventional open thyroidectomy is the standard treatment for PTMC, but is associated with certain risks that can affect patients’ health and quality of life.^2^ Over recent decades, AS has taken on an important role in the management of these tumours, providing a safe and feasible alternative. This strategy is based on close monitoring and early recognition of the minority group of patients who will develop clinical progression and will benefit from deferred surgery, thus optimising therapeutic resources and minimising adverse events.^3^ However, patients’ fear of cancer diagnosis and the anxiety due the risk of future metastases lead them to seek alternative options.

Percutaneous, energy-based ablation technologies such as RFA and MWA were introduced to clinical practice in the early 2000s. Thermal ablation was initially approved in the management of liver, pancreas and adrenal tumours, whereas their application on benign thyroid nodules was proposed in 2002.^5^ In 2014, the first study of MWA trial on PTMC was published,^9^ with the first outcomes of using RFA on PTMC reported two years later.^11^ Since then, the rapidly increasing number of published studies demonstrates the notable growth of interest in the field of minimaly ablative methods for PTMC management.

Although the technique followed during the procedure was similar in almost all studies, hydrodissection was an important additional step performed in many institutions. It was considered as highly important by some authors and was performed before ablation to keep a safe distance between the thyroid nodule and critical anatomical structures such as the carotid sheath during the procedure, aiming to minimise the risk of complications.

Both MWA and RFA seem to achieve satisfactory efficacy and safety in the management of low-risk PTMC, and could be a potential alternative option versus the stressful AS choice and high-cost, complication-related surgical intervention. However, any conclusions should be drawn with caution.

Our review reveals similar outcomes concerning CDR, VRR and complication rates following thermal ablation compared with previous reviews and meta-analyses. The vast majority of included studies reported high volume reduction and CDR rates, with values over 80%. However, the wide range in the reported CDR (between 15.2% and 100%) is remarkable. Several reasons could potentially explain this finding, including variation in eligibility criteria for ablation, the different size of study populations, the duration of the follow-up period and the experience of the physician performing the procedure.

A review by Choi *et al* compared RFA, MWA and LA on PTMC treatment, with VRR reaching 90% in most of the studies, while having a complication rate of less than 0.4%.^6^ The meta-analysis demonstrated that although all ablation techniques were effective and safe for PTMC treatment, a significant heterogeneity in CDR was noted. Namely, compared with RFA and MWA, LA was less effective in reducing the volume of PTMC. Another meta-analysis demonstrated no difference in efficacy and safety between RFA, MWA and LA.^42^ However, the authors concluded that RFA is superior to MWA and LA in terms of operation time, complication rates and lymph node metastases. On the other hand, MWA showed an advantage compared with the other ablation techniques with regard to risk of recurrence, while the LA group had the longest hospital stays.

A significantly better quality of life in patients with PTMC following MWA or RFA ablation compared with surgery was demonstrated, which is consistent with previously published reviews.^6,42^ Duration of procedure, blood loss, hospital stay and treatment costs were all significantly higher in the surgery group.^10,13,23,27,43^ Moreover, severe complications such as recurrent laryngeal nerve palsy and hypocalcaemia were noted in the surgical group, whereas thermal ablation caused mild transient symptoms. A comparison of 339 PTMC patients undergoing thermal ablation and 339 PTMC patients undergoing surgery for low-risk PTMC revealed that thermal ablation had the advantage of achieving a lower complication rate.^44^ In addition, although anxiety seems to be a major reason that patients with low-risk PTMC choose surgery over AS, it does not seem to affect those selecting thermal ablation.^44^

In contrast, the ablation group carries a small risk of PTMC recurrence, with possible responsible factors including larger initial tumor volume and nodule location closer to the capsule.^37^ According to our study, PTMC recurrence and LNM rates were 1% and 0.7%, respectively, whereas no cases with distant metastases were reported. Our results are similar to those reported in a previous review demonstrating that 0.2% of patients developed recurrent PTMC, whereas 0.4% of patients experienced LNM.^45^ Even in the case of PTMC recurrence, an additional ablative session seemed to be effective, with no residual tumor tissue detected at follow-up. Nevertheless, the wide range of follow-up period on each study should be noted.

Considering the above advantages, thermal ablation may be considered as an alternative treatment option for low-risk PTMC in patients who refuse surgery and AS or are ineligible for surgery. Nevertheless, despite the encouraging results, it is important to underline the existing risk for PTMC recurrence or LNM. For that reason, all patients should undergo long-term close monitoring including imaging (±FNA), aiming for early detection of tumour recurrence or LNM, as selected patients will benefit from either an additional ablation session or rescue surgery. A challenge in monitoring patients postablation compared with those undergoing surgery is the lack of measurable margins of the ablated nodule. It is difficult to evaluate changes and define whether the tumour has been completely destroyed or residual tissue exists with a risk for recurrence.

Thermal ablation for primary low-risk PTMC is a relatively new treatment modality and, for that reason, most studies include a relatively small number of patients with short follow-up compared with available data related to surgery and AS. Moreover, randomised controlled studies have not yet been performed. For that reason, a reliable comparison between those therapeutic options for low-risk PTMC cannot be made.

Our study has some limitations. First, only two databases, Pubmed and Cochrane, were used during the search process. Second, most studies included in the review were conducted in China with the same population demographics; the results could be different in other parts of the world, particularly since there is limited research on the subject in Europe or the United States. Third, different studies were conducted by the same team of researchers and some may have overlapping samples in retrospective work. Moreover, there were only few studies with follow-up duration greater than five years and only five comparative studies of thermal ablation of PTMC with surgery. Finally, all included studies were subject to moderate-to-high risk of bias.

## Conclusion

In conclusion, our study demonstrates that ultrasound-guided RFA and MWA are safe and effective minimally invasive methods for the treatment of low-risk PTMC patients as an alternative to surgery and/or AS. Thermal ablation under local anaesthesia could also be the key therapeutic option for patients who refuse, or are not willing to undergo, thyroidectomy. It is a highly promising and cost-effective procedure for the healthcare system, associated with short hospital stay and low morbidity. Nevertheless, further long-term, high-quality, randomised and multicentre trials with higher numbers of patients are required to confirm the safety and cost-effectiveness of this minimally invasive modality in the management of low-risk PTMC.
